# Organelle Genomes of *Epipogium roseum* Provide Insight into the Evolution of Mycoheterotrophic Orchids

**DOI:** 10.3390/ijms25031578

**Published:** 2024-01-27

**Authors:** Zhuang Zhao, Yuanyuan Li, Jun-Wen Zhai, Zhong-Jian Liu, Ming-He Li

**Affiliations:** Key Laboratory of National Forestry and Grassland Administration for Orchid Conservation and Utilization at College of Landscape Architecture and Art, Fujian Agriculture and Forestry University, Fuzhou 350002, China; fafuzzhuang@163.com (Z.Z.); lyy9902140575@163.com (Y.L.); zhai-jw@163.com (J.-W.Z.)

**Keywords:** high-throughput sequencing, multichromosomal mitogenome, mycoheterotrophic plants, organelle genomes, phylogenetic analysis

## Abstract

*Epipogium roseum*, commonly known as one of the ghost orchids due to its rarity and almost transparent color, is a non-photosynthetic and fully mycoheterotrophic plant. Given its special nutritional strategies and evolutionary significance, the mitogenome was first characterized, and three plastomes sampled from Asia were assembled. The plastomes were found to be the smallest among Orchidaceae, with lengths ranging from 18,339 to 19,047 bp, and exhibited high sequence variety. For the mitogenome, a total of 414,552 bp in length, comprising 26 circular chromosomes, were identified. A total of 54 genes, including 38 protein-coding genes, 13 tRNA genes, and 3 rRNA genes, were annotated. Multiple repeat sequences spanning a length of 203,423 bp (45.47%) were discovered. Intriguingly, six plastid regions via intracellular gene transfer and four plastid regions via horizontal gene transfer to the mitogenome were observed. The phylogenomics, incorporating 90 plastomes and 56 mitogenomes, consistently revealed the sister relationship of *Epipogium* and *Gastrodia*, with a bootstrap percentage of 100%. These findings shed light on the organelle evolution of Orchidaceae and non-photosynthetic plants.

## 1. Introduction

Mycoheterotrophic plants have long fascinated botanists and mycologists due to their unique ability to obtain carbohydrates from fungi rather than through photosynthesis [1,2]. In the Orchidaceae family, the seeds are characterized by their small, dust-like size and lack of endosperm. Consequently, mycoheterotrophy is a common phenomenon during the germination stage of their life cycle [3], and most species continue to derive carbohydrates from fungi even after they have developed the capacity for photosynthesis [4,5,6]. However, some orchids, such as *Epipogium*, *Gastrodia*, and *Rhizanthella*, fully rely on fungal nutrients throughout their entire life cycle, making them invaluable resources for studying the occurrence and evolution of mycoheterotrophy.

Plastids and mitochondria play crucial roles in plants. The plastid genome (plastome) generally exhibits a stable genome structure and size, making it more amenable to sequencing and assembly [7]. In contrast, the mitochondrial genome (mitogenome) exhibits significant variation in size and structure among species and even populations [8,9]. The availability of substantial evolutionary information in organelle genomes has contributed to their widespread use in phylogeny and evolutionary biology studies. To date, there have been 11,634 complete plastomes and 593 mitogenome deposited in the NCBI Organelle Genome Resources database [10]. Orchidaceae is one of the largest families of angiosperms [11]. More than 900 plastomes of Orchidaceae have been reported, while only six mitogenomes have been published, including four autotrophic species (*Apostasia shenzhenica*, *Cymbidium lancifolium*, *C. macrorhizon*, and *Paphiopedilum micranthum*) and two fully mycoheterotrophic species (*Gastrodia elata* and *G. pubilabiata*) [12,13,14,15,16].

Intercellular gene transfers (IGTs) occurring frequently among the nucleus, plastids, and mitochondria enhance genetic material movement within organisms [17]. This process enables the nucleus to exert control over the organelles through encoding organelle-specific proteins and tRNA genes. Horizontal gene transfers (HGTs) have played a significant role in the evolution of eukaryotic genomes. The number of well-supported cases of HGTs with functional implications is rapidly expanding, highlighting their importance in niche adaptation and their varying impact across different lineages [18]. Plant mitogenomes exhibit diverse structures characterized by multiple rearrangements. In addition, IGTs and HGTs have been reported in plant mitogenomes, further complicating the prediction of their characteristics [19,20,21].

The fully mycoheterotrophic orchid *Epipogium roseum* (D.Don) Lindl., commonly known as one of the ghost orchids due to its rarity, almost transparent color, and underground life history except during flowering, is the focus of this study. *E. roseum* primarily grows on densely shaded forest floors and exhibits an obligate self-pollination system [22]. It is widely distributed in tropical and subtropical regions of Asia, Australia, and Africa. Previous research has reported and discussed five *E. roseum* plastomes from Cameroon, Vanuatu, and Vietnam [23]. The results revealed several unique characteristics of *E. roseum*, including possessing the smallest plastome (ca. 19 kb) within the Orchidaceae, as well as exhibiting intraspecific plastome polymorphism, multiple rearrangements, highly biased nucleotide composition, and an unprecedentedly high substitution rate. The morphology, reproductive patterns, and plastome evolution of *E. roseum* have undergone investigation. However, the plastome from Asia and the mitogenome remain unexplored and unreported to date.

In this study, three plastomes from Asia and the first complete mitogenome of *E. roseum* were sequenced, assembled, and annotated, aiming to address several key questions: (1) How do plastomes vary across different regions? (2) What are the characteristics of the mitogenome? (3) Are there gene transfer events existing between organelle genomes?

## 2. Results

### 2.1. Characterization and Comparative Analysis of Plastomes

In this study, three complete plastomes from Asia were newly assembled and annotated, as presented in Supplementary Table S1. These plastomes exhibited extreme reduction, being the smallest known among orchids, with lengths ranging from 18,339 to 19,047 bp and low GC contents (30.03–30.96%). Each plastome comprised two single-copy (SC) regions and two considerably contracted inverted repeat (IR) regions (198–292 bp). The length of the SC1 regions ranged from 8326 bp to 8911 bp, with a GC content of 23.14% to 24.49%. The SC2 regions spanned from 9427 bp to 9618 bp, with a GC content of 35.93% to 36.84% (Supplementary Figure S1, Supplementary Table S6). A total of 29 common genes, including 18 protein-coding genes (PCGs), 7 tRNA genes, and 4 rRNA genes, were annotated in the plastomes (Supplementary Figure S1, Supplementary Table S1).

**Figure 1:**
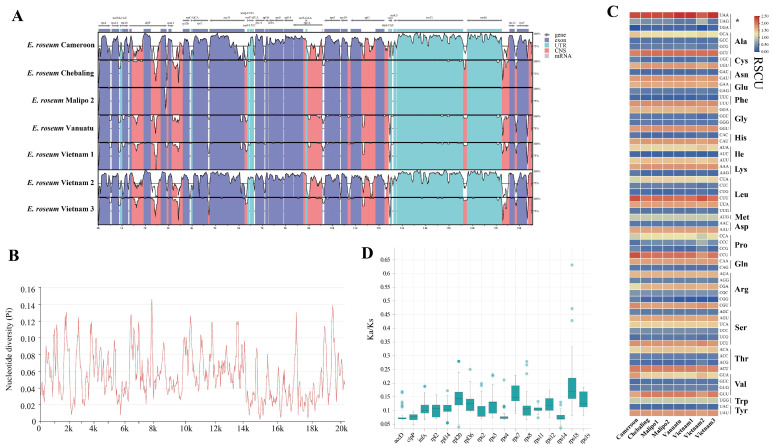
Comparative analyses of eight *E. roseum* plastomes. (**A**) Sequence alignment of the plastomes. Thick, gray arrows above the alignment indicate the orientation and position of each gene; the Y-axis represents the identity percentage, ranging from 50 to 100%. (**B**) The nucleotide diversity of plastomes using a sliding window test. The window size was set to 100 bp, and the sliding window size was 25 bp. X-axis, position of the midpoint of a window; Y-axis, Pi values of each window. (**C**) The RSCU values of 18 concatenated protein-coding genes for the plastomes. * indicates stop codon. The red values mean higher RSCU, and the blue values mean lower RSCU. (**D**) The boxplots of Ka/Ks values among 17 PCGs in plastomes with five orchids. The “X” axis shows the names of protein-coding genes, and the “Y” axis shows the Ka/Ks values.

To explore the divergence of complete sequences among *E. roseum* plastomes with increased sampling, comparative analyses were conducted. Visual alignment results (Figure 1A) and nucleotide diversity (Pi) calculations (Figure 1B) illustrated high intraspecific plastome polymorphism among these sequences. Due to their distinctive features, the plastome of sample “Malipo 1” was selected as the reference sequence. The sequence of sample Malipo 2 exhibited the highest similarity, and Cameroon and Vietnam 2 displayed the highest polymorphism. *E. roseum* plastomes showed high Pi values ranging from 0.003 to 0.146 (Figure 1B, Supplementary Table S2). Although the plastomes shared common PCGs, the codons of these genes and the RSCU varied (Figure 1C, Supplementary Table S3). Furthermore, non-synonymous substitutions (Ka) and synonymous substitutions (Ks) were calculated for the 17 PCGs in *E. roseum* plastomes, along with five other orchids. The Ka/Ks ratios for all PCGs were found to be much lower than 1, indicating the stability of protein function for these genes during evolution (Figure 1D, Supplementary Table S4).

### 2.2. Comprehensive Analysis of Mitogenome Characteristics

The mitogenome of *E. roseum* was assembled into 26 circular chromosomes, with lengths ranging from 9865 to 23,000 bp, resulting in a total length of 414,552 bp (Table 1, Supplementary Figure S2). The average GC content of the *E. roseum* mitogenome was 45.2%, with individual chromosomes ranging from 41.6% to 48.0% in GC content. It contained a total of 54 genes, including 38 protein-coding genes (PCGs), 13 tRNA genes, and 3 rRNA genes (*rrn5*, *rrn18*, and *rrn26*). The PCGs comprised five ATP synthase genes (*atp1*, *atp4*, *atp6*, *atp8*, and *atp9*), nine NADH dehydrogenase genes (*nad1*, *nad2*, *nad3*, *nad4*, *nad4L*, *nad5*, *nad6*, *nad7*, and *nad9*), four cytochrome c biogenesis genes (*ccmB*, *ccmC*, and *ccmFN*), three cytochrome c oxidase genes (*cox1*, *cox2*, and *cox3*), one Maturases gene (*matR*), one membrane transport protein gene (*mttB*), and one ubichinol cytochrome c reductase gene (*cob*). Six PCGs, namely *nad1*, *nad2*, *nad5*, *nad7*, *ccmFc*, and *rpl2*, were either incomplete or pseudogenized. Among the genes, *nad2* and *rps10* contained one intron each, *cox2* contained two introns, and *nad4* contained three introns. Additionally, the tRNA genes *trnE-TTC* and *trnM-CAT* were present in two copies.

**Figure 2:**
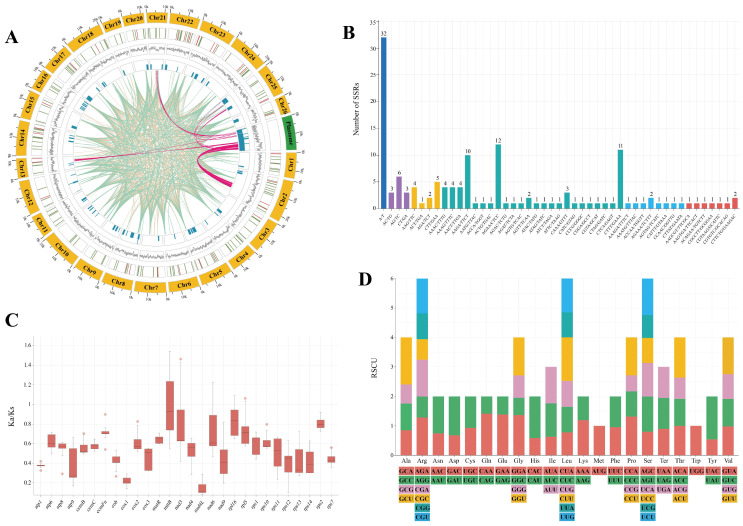
Comparative analysis of *E. roseum* mitogenome. (**A**) Features of mitogenome tabulated in a Circos plot. The circles from outside to inside represent lengths of mitochondrial contigs and plastome; positions of SSRs (colored red) and tandem repeats (colored green); GC ratios; gene density; homologous fragments among plastome and mitogenome (colored red); forward repeats (colored blue); and palindromic repeats (colored yellow). (**B**) SSR distribution of mitogenome. (**C**) Ka/Ks ratios of mitogenome PCGs. (**D**) RSCU of mitogenome PCGs.

A total of 139 simple sequence repeats (SSRs) comprising 1- to 6-nucleotide motifs were identified in the mitogenome of *E. roseum* (Figure 2A,B; Supplementary Table S5). Among these SSRs, tetranucleotide repeats were the most abundant, accounting for 47.48% of the total, and pentanucleotide and hexanucleotide repeats had nine occurrences each. Mononucleotide repeats were exclusively composed of A/T SSRs, and dinucleotide and tetranucleotide repeats were observed 12 times each. The most frequently observed repeat motif was the A/T mononucleotide, occurring 32 times (Figure 2B, Supplementary Table S5). Furthermore, the *E. roseum* mitogenome contained 47 tandem repeats, all with a 100% match. These repeats ranged in length from 25 to 150 bp (Figure 2A, Supplementary Table S6). Additionally, 997 dispersed repeats were identified in the *E. roseum* mitogenome, consisting of 511 forward repeats and 486 palindromic repeats (Figure 2A, Supplementary Table S7).

To explore the selection pressure on the mitochondrial PCGs, the non-synonymous substitution rate (Ka) and synonymous substitution rate (Ks) with five other orchids—*Apostasia shenzhenica*, *Cymbidium lancifolium*, *Dendrobium amplum*, *Gastrodia pubilabiata*, and *Paphiopedilum micranthum*—were separately calculated (Figure 2C, Supplementary Table S9). Overall, the Ka/Ks ratios among these orchids were generally similar, with the majority of ratios being less than 1. However, we observed Ka/Ks ratios greater than 1 for the *nad6* gene with *A. shenzhenica*, the *rpl16* gene with *D. amplum*, the *nad3* gene with *G. pubilabiata*, and the *mttB* and *rpl5* genes with *P. micranthum* (Figure 2C, Supplementary Table S8).

A codon preference analysis was performed on 30 PCGs and 6828 codons in the *E. roseum* mitogenome (Figure 2D, Supplementary Table S9). Among these codons, stop codons were observed 40 times. The most frequently occurring amino acid was leucine (Leu), appearing 696 times, while cysteine (Cys) was the least frequent, occurring only 90 times. Analysis of the relative synonymous codon usage (RSCU) revealed that GCU and CUU had the highest codon usage bias (CUB), with values of 1.589 and 1.470, respectively. On the other hand, UAC and CCG had the lowest CUB, with values of 0.538 and 0.545, respectively. Out of the codons analyzed, 28 exhibited RSCU values greater than 1, while 34 had values below 1. The RSCU values for the codons AUG (encoding methionine, Met) and UGG (encoding threonine, Thr) were both determined to be 1.

### 2.3. Migration of Plastid Regions in Mitogenome

To investigate intracellular gene transfers (IGTs), a homologous fragment analysis was performed between *E. roseum* mitogenome and two plastomes that had sequence differences (sample Malipo 1 and Vietnam 2). The analysis revealed the presence of homologous regions, with sample Malipo 1 having 4549 bp and Vietnam 2 having 6513 bp of homologous regions (Supplementary Table S10). Within these homologous regions, certain plastid genes were identified, including *accD*, *clpP*, *rpl2*, *rps2*, *rrn16*, and *rrn23* genes. However, it was observed that the majority of these regions were rRNA regions, while the coding gene regions were too short for further alignment or comparison.

**Figure 3:**
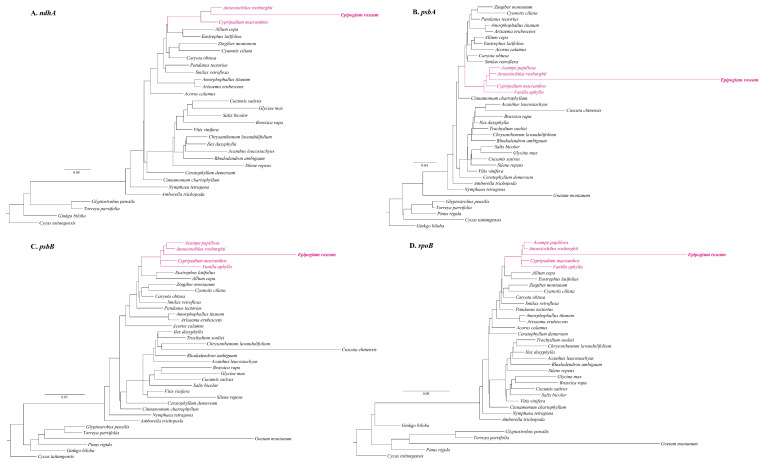
A maximum likelihood (ML) phylogenetic tree of four regions found to be plastid-origin coding regions in the mitogenome. The numbers near the nodes are bootstrap percentages. The position of Orchidaceae based on mitogenome is highlighted in red, and position of *E. roseum* is bold.

A database of 5366 published plastomes of vascular plants was established to search for additional plastid regions in the *E. roseum* mitogenome. To focus on coding gene regions, the plastome database was curated by removing intergenic spacer regions and rRNA and tRNA genes. The analysis revealed that the *E. roseum* mitogenome had 37,008 blast hits against the plastome database (Supplementary Table S11). Based on the continuity of blast hits and the length of the plastid regions in the mitogenome, four distinct blocks, namely *nadA*-like, *psbA*-like, *psbB*-like, and *rpoB*-like, were identified as representatives for further alignment and phylogenetic analysis (Figure 3). Alignments and phylogenetic analyses were performed on these four blocks, comparing them with related genes in 36 other plant plastomes to determine their phylogenetic positions. The results demonstrated that all four plastome-related sequences nested within the Orchidaceae family and plastome-related regions displayed long branches in the phylogenetic topology (Figure 3).

### 2.4. Phylogenetic Position of E. roseum

**Figure 4:**
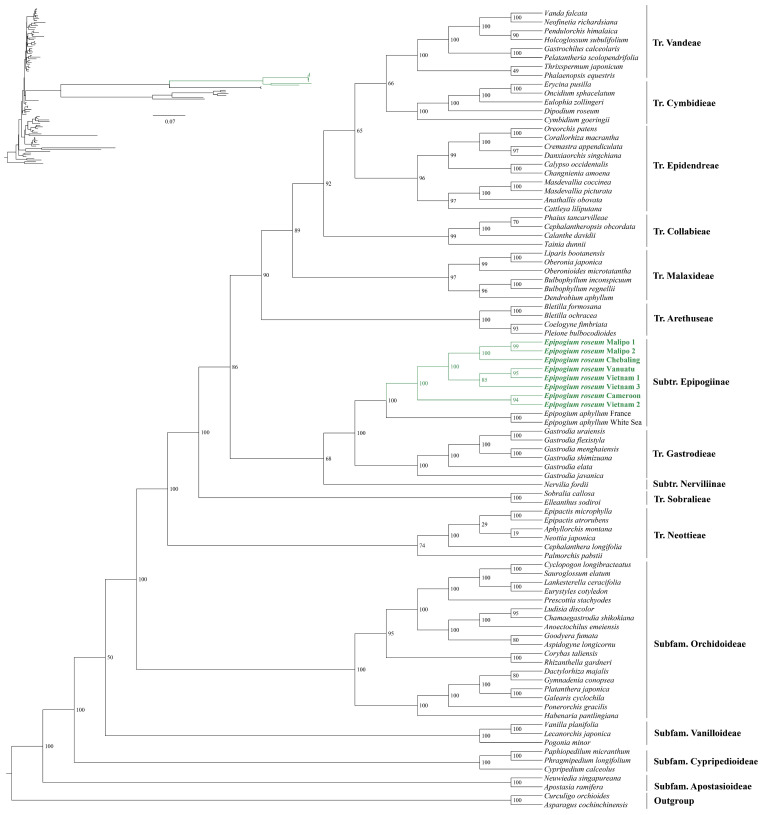
A maximum likelihood (ML) phylogenetic tree of 90 plastomes based on 14 PCGs. The numbers near the nodes are bootstrap percentages; upper left corner shows the phylogenetic topology structure. The position of *E. roseum* is highlighted in green and bold.

The phylogenetic analysis was conducted using 14 PCGs from the plastomes of 88 Orchidaceae species along with two outgroup species (*Curculigo orchioides* and *Asparagus cochinchinensis*) (Figure 4, Supplementary Table S12). The alignment matrix comprised 10,919 bp, with 5643 variable sites and 4236 parsimony informative sites. The resulting phylogenetic tree displayed several long branches, including *Epipogium*, *Gastrodia*, *Rhizanthella gardneri*, *Lecanorchis japonica*, and *Vanilla planifolia*. These long branches primarily consisted of mycoheterotrophic orchids, except for *V. planifolia*, which may be closely related to *L. japonica*. The phylogenetic analysis confirmed the monophyly of the genus *Epipogium*, consisting of two monophyletic groups: *E. aphyllum* and *E. roseum*. Furthermore, the analysis revealed the relationships among the eight samples of *E. roseum* as follows: (Cameroon, Vietnam 2), (Vietnam 3, (Vietnam 1, Vanuatu)), (Chebaling, (Malipo 1, Malipo 2)). Based on branch lengths, *E. roseum* can be divided into two clades: one clade comprising Cameroon and Vietnam 2, and the other clade comprising the other samples.

**Figure 5:**
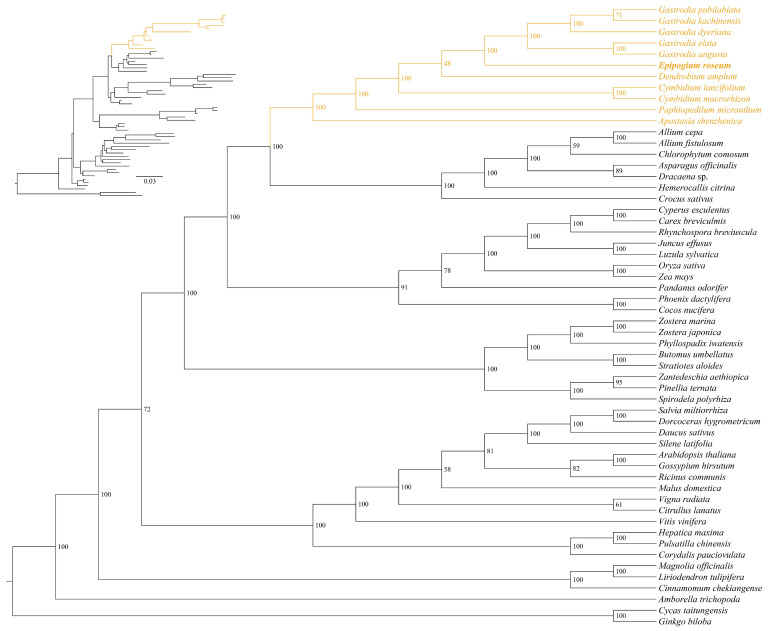
A maximum likelihood (ML) phylogenetic tree of 56 mitogenomes based on 38 PCGs. The numbers near the nodes are bootstrap percentages; upper left corner shows the phylogenetic topology structure. The position of Orchidaceae based on mitogenome is highlighted in yellow, and position of *E. roseum* is bold.

In the mitochondrial phylogenetic analysis, 30 conserved mitochondrial protein-coding genes (PCGs) were analyzed from a total of 56 vascular plants (Figure 5, Supplementary Table S12). The mitogenomes of *Ginkgo biloba* and *Cycas revoluta* were used as outgroups for comparison. The alignment matrix used for analysis comprised a total of 50,220 bp, with 19,775 variable sites and 11,404 parsimony informative sites. The phylogenetic tree showed that *E. roseum* was closely related to *Gastrodia* with robust support (Figure 5). Additionally, the mitogenomes of Orchidaceae family shared one clade and comprised a monophyletic group. The relationships among the mitogenomes of Orchidaceae are as follows: (*Apostasia shenzhenica*, (*Paphiopedilum micranthum*, (*Cymbidum*, (*Dendrobium amplum*, (*Epipogium roseum*, *Gastrodia*))))). This analysis provides valuable insights into the evolutionary relationships and genetic connections among Orchidaceae species and other vascular plants based on their mitogenomes.

## 3. Discussion

The newly assembled plastomes of *E. roseum* exhibited structural characteristics, sequence length, gene content, and GC content that fell within the range reported for previous plastomes [23]. The comparative genomic results revealed unusual structure, genome reduction, and high intraspecific polymorphism in *E. roseum* plastomes. Most plastomes within the Orchidaceae family displayed the typical quadripartite structure, consisting of a large single-copy (LSC) region, a small single-copy (SSC) region, and two inverted repeat (IR) regions [24,25]. In contrast, the plastomes of *E. roseum* had two small IR regions, ranging from 198 to 292 bp, and two single-copy (SC) regions. Within the smaller SC region, genes such as *accD* and *clpP* shared similarities with the LSC regions of other Orchidaceae plastomes; genes within the larger SC region, particularly the four rRNA genes, exhibited similarity to the IR regions of other Orchidaceae plastomes. Previous studies reported nucleotide diversity values (Pi) of 0.14 for five *Epidendrum* plastomes and 0.129 for seven *Trichoglottis* plastomes [26,27]. In the case of *E. roseum*, the high intraspecific plastome polymorphism is manifested by Pi values reaching up to 0.146. Additionally, the relative synonymous codon usage (RSCU) values were found to vary among different populations.

In this study, the complete mitogenome of *E. roseum* was reported for the first time. It was found to have 26 circular chromosomes with a total length of 414,552 bp. Previous studies have explored the complex structures and varying genome sizes of plant mitogenomes [9,15,28]. Many orchid mitogenomes published before were multichromosomal, including species like *Cymbidium lancifolium*, *Cymbidium macrorhizon*, *Gastrodia elata*, *Gastrodia pubilabiata*, and *Paphiopedilum micranthum*, with total lengths ranging from 447,368 bp (*P. micranthum*) to 1,340,105 bp (*G. elata*). Hence, the multichromosomal structure observed in *E. roseum* seems reasonable. A total of 54 genes, including 38 protein-coding genes (PCGs), 13 tRNA genes, and 3 rRNA genes, were successfully annotated. However, some PCGs were incomplete or showed pseudogenization. Some genes contained multiple introns that spanned several chromosomes, such as *nad1*, *nad2*, *nad5*, and *nad7*, which posed challenges for perfect assembly and annotation. The *ccmFc* gene exhibited significant sequence similarity with other orchids, but the CDS could not be successfully annotated due to unexpected stop codons.

Sequences with repetitive elements have been shown to facilitate various rearrangements in plant mitogenomes [29]. Repeat-mediated recombination has been observed in plant mitogenomes, including those of *P. micranthum*, *Ipomoea batatas*, and *Gelsemium elegans* [15,19,29]. In this study, we identified tandem repeats, dispersed repeats, and SSRs within *E. roseum* mitogenome, with a total length of 203,423 bp (45.47%). However, the potential for recombination and the hypothesis of a master circle were not further explored. To gain insights into the evolution of PCGs in *E. roseum* organelle genomes, Ka/Ks analysis was performed. Despite the loss of multiple genes in the plastomes, the lower Ka/Ks ratios indicated the stability of the remaining PCGs. The Ka/Ks ratios observed in both plastid and mitochondrial PCGs indicated a consistent pattern of negative selection and their evolutionary significance.

The presence of plastome-derived sequences in plant mitogenomes can be attributed to IGTs and horizontal gene transfers (HGTs). For instance, in *Paphiopedilum micranthum* (Orchidaceae), 59% of the plastome-origin mitochondrial sequences were identified [15]. In *Vitis vinifera* (Vitaceae), it was 8.8% [30], and in *Actinidia* (Actinidiaceae), it ranged from 1.16% to 4.05% [31]. Photosynthetic plants often exhibit plastome-origin mitochondrial sequences through IGTs occurring between plastomes and mitogenomes themselves [15,20,32]. Notably, the identification of HGTs appears to be more significant in non-photosynthetic plants, leading to further research on foreign plastid sequences in plant mitogenomes [16,33,34]. To investigate the IGTs in the *E. roseum* mitogenome, two representative samples were utilized, considering the sequence variations in plastomes. The analysis resulted in the identification of six plastid regions with lengths of 4549 and 6513 bp. However, the coding gene regions were too short for meaningful alignment or comparison. Consequently, an exploration of homologous regions among a database of plant plastomes and mitogenomes was carried out. Through alignment and phylogenetic analysis, four plastid regions transferred via HGTs were confirmed.

The phylogenetic relationships of *Epipogium* have been a subject of intense debate in orchid systematics [35]. Different studies using various genetic markers have proposed conflicting placements for *Epipogium*. For instance, an analysis based on nuclear ITS suggested a close relationship between *Epipogium* and Nervilieae [36], while another study indicated it to be sister to Triphoreae. On the other hand, a phylogenetic analysis using mitochondrial nad1 sequences strongly supported *E. aphyllum* as the sister species to *Nervilia shirensis* [35]. In a larger-scale study involving 1450 low-copy nuclear genes from 610 orchid species, the phylogeny indicated *Epipogium* to be sister to *Nervilia* with moderate support [37]. Consequently, these studies led to the placement of *Epipogium* within the tribe *Nervilieae* and the subtribe *Epipogiinae*. In this particular study, phylogenetic trees using 14 PCGs from 90 plastomes and 30 PCGs from 56 mitogenomes were reconstructed. The results supported *Epipogium* being sister to *Gastrodia* with 100% bootstrap percentages for both plastid and mitochondrial datasets.

This study reports three *E. roseum* plastomes and conducts a comparative analysis to reveal their sequence variety. The mitogenome was sequenced and annotated, followed by a comprehensive analysis of genome characteristics. IGTs and HGTs were identified by sequence similarity and phylogeny. Morever, system evolution analysis was conducted to comprehend the phylogenetics of it. This research provides a theoretical foundation for exploring the organelle genomes of mycoheterotrophic orchids.

## 4. Materials and Methods

### 4.1. Sample Sampling, DNA Extraction and Sequencing, Genome Assembly and Annotation

Plant samples were collected from Guangdong Chebaling National Nature Reserve (Shixing County, China) and Malipo County (China) (Supplementary Table S1) and stored at the herbarium of the College of Forestry, Fujian Agriculture and Forestry University (FJFC). Genomic DNA was extracted from fresh tubers, stems, and flowers using a modified CTAB protocol [38]. Short reads were sequenced using an Illumina HiSeq 4000 platform (San Diego, CA, USA). To construct genomic libraries (SMRTbell libraries) for PacBio long-read sequencing, high-molecular-weight genomic DNA was sheared into fragments of approximately 20 kb. Then, large-fragment genomic DNA was concentrated with AMPure PacBio beads and used for SMRTbell preparation according to the manufacturer’s specifications (Pacific Biosciences, Menlo Park, CA, USA). The libraries were constructed and sequenced using the PacBio Seque II sequencing platform (Pacific Biosciences, Menlo Park, CA, USA).

Plastome assembly and annotation were conducted following the methodologies described in our previous study [39]. The short reads were de novo assembled into the mitogenome using GetOrganelle v1.7.6.1 [40]. Upon assembly, the original contigs exhibited a pattern of ‘fireworks’, with a length of 437,794 bp (Supplementary Figure S3). The visualization of this assembly was conducted using Bandage v0.8.1 [41]. Subsequently, the long reads were filtered utilizing BLASR v5.1 [42], using the original assemblies as a reference. The filtered long reads were then assembled using CANU v1.8 [43]. The resulting contigs obtained from CANU assembly were considered as the reference, and the assembly process was iterated until stable contigs were obtained. Finally, the circular nature of the contigs was manually determined through overlap analysis.

For gene annotation, the coding and rRNA genes were annotated in Geneious Prime (Biomatters, Inc., Auckland, New Zealand), employing published orchid mitogenomes as references. These annotations were then manually refined. Furthermore, tRNA genes were annotated using tRNAscan-SE v2.0 [44]. To visualize the genome maps, Organellar Genome DRAW [45] was employed.

### 4.2. Genome Characters and Comparative Genomics

To analyze the diversity of *E. roseum* plastome sequences, the online program mVISTA was employed, specifically utilizing the Shuffle-LAGAN alignment program [46]. The protein-coding genes (PCGs) were extracted using PhyloSuite v1.2.2 [47] and aligned using MAFFT 7 [48]. The nucleotide variability (Pi) for the eight plastomes was calculated using DnaSP 6 [49], with a window length of 100 bp and a step size of 25 bp. The relative synonymous codon usage (RSCU) was analyzed using DAMBE 7 [50]. Furthermore, the Ka/Ks ratios were calculated using KaKs_Calculator2 [51].

To identify tandem repeats, the Tandem Repeats Finder v4.09 program [52] was employed with default parameters. The REPuter [53] was used with default parameters to detect four types of long repeats, namely forward (F), palindrome (P), reverse (R), and complement (C) repeats. SSRs were identified using the Perl script MISA [54], with the minimum thresholds for mono-, di-, tri-, tetra-, penta-, and hexa-motif microsatellites set at 10, 5, 4, 3, 3, and 3 nucleotide repeats, respectively. ChiPlot Online Tools was used to visualize the obtained results [55].

### 4.3. Gene Transfer and Phylogenetic Analysis

The plastomes and mitogenomes used in this study were obtained from the NCBI Organelle Genome Resources database, and a summary of the sources can be found in Supplementary Table S11. To analyze sequence similarity between the plastomes and mitogenome and detect transferred DNA fragments, BLASTN [56] was utilized with an e-value cut-off of 1 × 10^−5^. For visualization purposes, the Circos module implemented in TBtools [57] was employed to generate a circle diagram.

A total of 90 plastomes (two outgroup species: *Curculigo orchioides* and *Asparagus cochinchinensis*) and 56 mitogenomes (two outgroup species: *Ginkgo biloba* and *Cycas revoluta*) were selected to conduct phylogenetic analysis. Supplementary Table S12 provides details of the taxa. To elucidate the phylogenetic analysis, maximum likelihood (ML) analysis was conducted using the RAxML-HPC2 on XSEDE 8.2.10 tool available on the CIPRES Science Gateway web server. The GTRCAT model was specified for all datasets, and 1000 repeated self-expanding analyses were performed [58].

## 5. Conclusions

In this study, we present the first complete mitogenome of the fully mycoheterotrophic orchid *Epipogium roseum*. Additionally, three plastomes sampled from China were assembled and analyzed. Our findings shed light on the intraspecific plastome polymorphism, general characteristics of the mitogenome, and the phylogenetic position of *E. roseum*. The mitogenome of *E. roseum* exhibited multiple sequence repeats, comprising a total length of 203,423 bp (45.47%). Intracellular gene transfers (IGTs) and horizontal gene transfer (HGT) events were identified within organelle genomes. The phylogenomics supported the robust sister relationship between *Epipogium* and *Gastrodia*. Overall, this study adds to the understanding of organelle genomes in non-photosynthetic orchids and fully mycoheterotrophic plants.

## Figures and Tables

**Table 1:** Feathers of *E. roseum* mitogenome. * Genes with one intron. ** Genes with two introns. *** Genes with three introns. ψ Genes were incomplete or showed pseudogenization.

Chromosome	Length (bp)	GC Content (%)	Genes	PacBio Reads Coverage
Chr 1	14,127	42.5	*rps7*	22.27
Chr 2	23,000	45.8	ψ *atp4*, *atp8*, *nad4L*	22.24
Chr 3	17,940	44.3	*nad2* *	15.70
Chr 4	14,746	44.8	*psbE*, *trnQ*-*TTC*	10.66
Chr 5	17,464	46.2	*atp1*, *ccmFn*, ψ *nad5*	10.49
Chr 6	16,756	44.7	*atp9*	17.81
Chr 7	17,441	44.8	*rps2*	20.79
Chr 8	16,696	44.7	*atp6*, *cox2* **, ψ *nad3*, *rps11*, *trnM*-*CAT*	16.41
Chr 9	15,231	45.4	*cob*, ψ *nad1*, *rpl5*, *rps14*, *trnC-GCA*	12.31
Chr 10	20,234	46.6	*nad4* ***	20.48
Chr 11	12,241	45.2	ψ *nad5*	10.43
Chr 12	18,964	45.6	*nad3*, *rps12*, *trnE*-*TTC*, *trnH*-*GTG*	19.73
Chr 13	10,976	44.3	*trnD*-*GTC*, *trn*-*GTT*	14.64
Chr 14	19,264	46.4	*atp9*, *rps1*, *trnY*-*GTA*	22.61
Chr 15	15,510	45.8	*matR*	12.59
Chr 16	11,633	48.0	ψ *nad7*, *trnE*-*TTC*	6.56
Chr 17	15,703	46.0	ψ *rpl2*, *rpl16*, *rps19*	12.40
Chr 18	20,493	45.7	*mttB*, *rps13*	30.44
Chr 19	9973	44.7	*ccmB*	6.38
Chr 20	11,816	45.4	ψ *nad5*	5.82
Chr 21	11,460	41.6	*nad9*, *trnF*-*GAA*, *trnW*-*CCA*	15.88
Chr 22	16,388	44.8	*cox1*, *trnL*-*CAA*	15.47
Chr 23	19,053	45.0	*ccmC*, *nad6*, *rps10* *	21.72
Chr 24	20,245	45.3	*cox3*	21.19
Chr 25	17,333	45.1	*rrn5*, *rrn18*, *rrn26*, *trnM*-*CAT*	32.24
Chr 26	9865	45.4	ψ *ccmFc*	6.56

## Data Availability

The newly obtained complete mitogenome sequences that support the findings of the study have been deposited in the NCBI with accession numbers as follows: OR871626–OR871651; the accession numbers of plastome sequences were: OR871652–OR871654.

## Supplementary Materials

The following supporting information can be downloaded at: https://www.mdpi.com/article/10.3390/ijms25031578/s1.
