# Supplementary material for: Organelle Genomes of Epipogium roseum Provide Insight into the Evolution of Mycoheterotrophic Orchids
Source: Int J Mol Sci. 2024 Jan 27;25(3):1578. doi: 10.3390/ijms25031578 (PMC10855806; doi:10.3390/ijms25031578)
Supplement: Supplementary file 1 [file ijms-25-01578-s001.zip › Supplementary_Figures.pdf]

Supplementary Figures

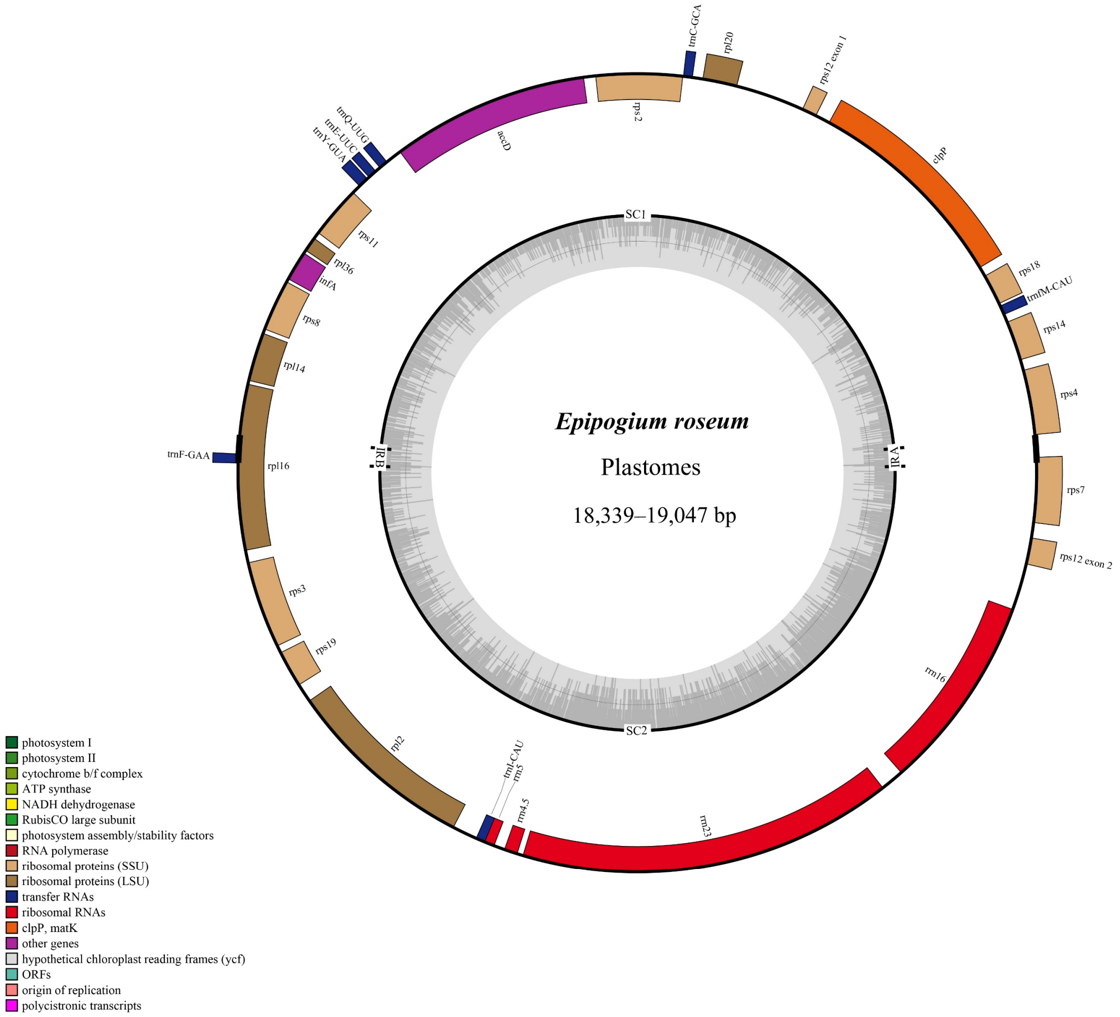

Supplementary Figure S1. The annotation maps of *Epipogium roseum* plastomes. The darker gray in the inner circle corresponds to GC content. The two inverted repeat and two single-copy regions are indicated outside of GC content.

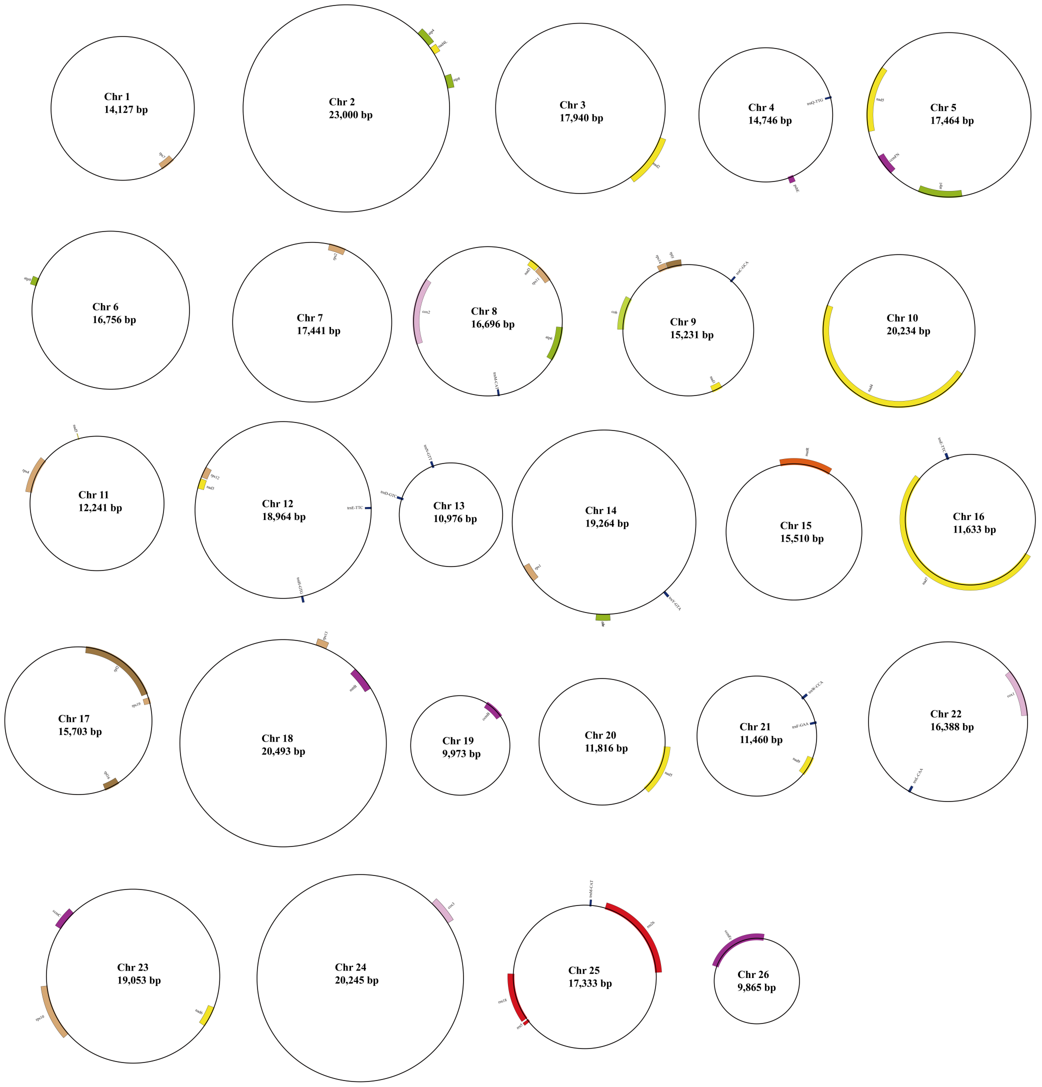

Supplementary Figure S2. The annotation maps of the *E. roseum* mitogenome.

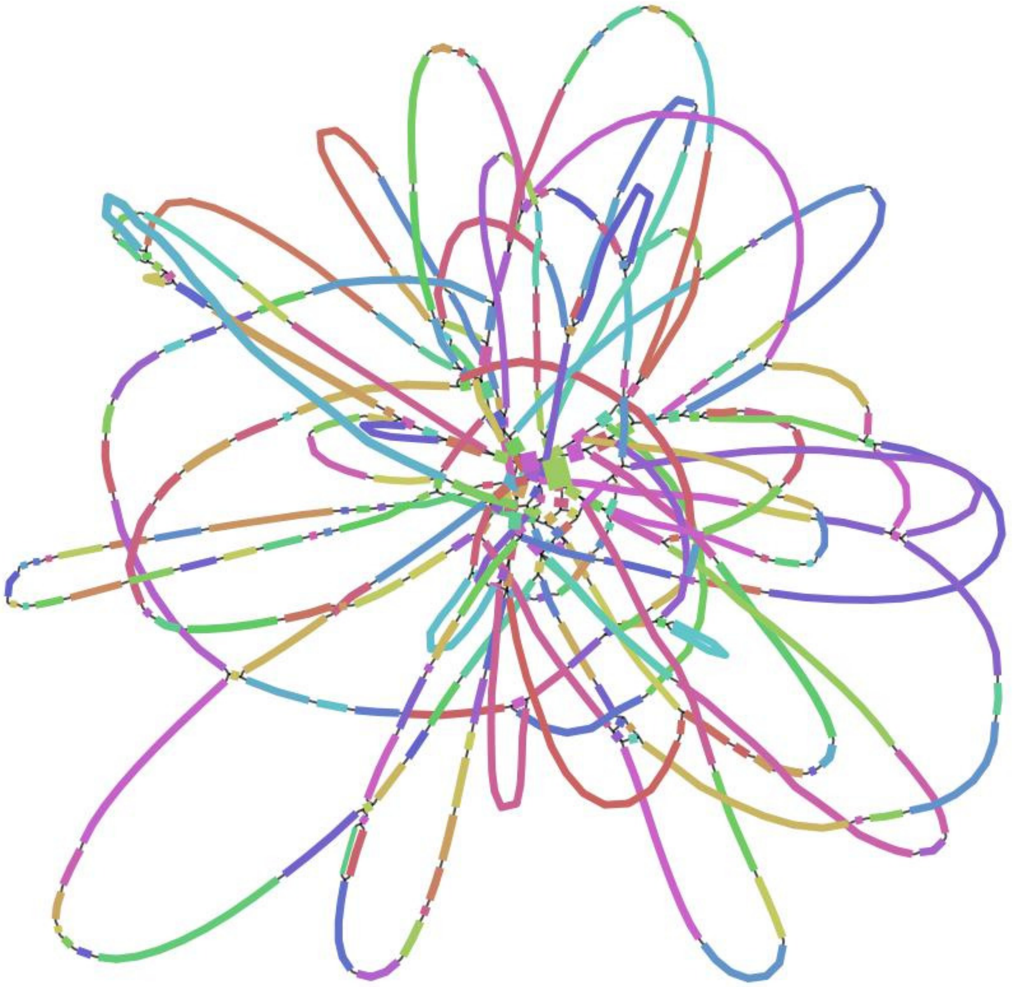

Supplementary Figure S3. The original contigs of *E. roseum* mitogenome exhibited a pattern of ‘fireworks’ used short reads.
